# Medications for Unhealthy Alcohol Use: Across the Spectrum

**Published:** 2011

**Authors:** Stephanie S. O’Malley, Patrick G. O’Connor

**Keywords:** Alcohol use, abuse and dependence, harmful drinking, hazardous drinking, treatment, primary care, pharmacotherapy, medication therapy, screening, counseling, brief intervention, continuum of care

## Abstract

The prevalence of unidentified or untreated unhealthy alcohol use remains high. With the advent of pharmacotherapy and models of counseling appropriate for use in primary care settings as well as in specialty care, clinicians have new tools to manage the range of alcohol problems across the spectrum of health care settings. By extending treatment to primary care, many people who do not currently receive specialty care may have increased access to treatment. In addition, primary care providers, by virtue of their ongoing relationship with patients, may be able to provide continuing treatment over time. Extending the spectrum of care to hazardous drinkers who may not be alcohol dependent could result in earlier intervention and reduce the consequences of excessive drinking.

Unhealthy alcohol use, which includes the spectrum of drinking behaviors and consequences ranging from risky use to problem drinking, along with alcohol abuse and alcohol dependence (Saitz 2005), has been linked to a multitude of health and social problems. Unhealthy alcohol use accounts for an estimated 85,000 deaths at an economic cost of $185 billion annually in the United States (Harwood 2000). Beyond this, numerous medical problems, such as liver disease, neurologic problems, and malignancies, as well as behavioral dysfunction resulting in employment and legal problems are directly attributable to alcohol.

Research has demonstrated that a variety of treatment approaches can help individuals with unhealthy alcohol use decrease their alcohol intake and thus avoid the many consequences described above. Counseling interventions have been designed to address the full spectrum of unhealthy alcohol use from brief interventions for risky use to more complex and rigorous counseling strategies for individuals with alcohol dependence. In addition, beginning with disulfiram in the late 1940s and more recently with naltrexone and acamprosate along with newer medications “in the pipeline,” pharmacotherapy has been demonstrated to be a useful adjunct to behavioral therapies for many people with unhealthy alcohol use, particularly those with alcohol dependence.

The spectrum of unhealthy alcohol use can be addressed in a variety of health care settings, including primary care, specialty practice, and alcohol treatment programs. Although complex behavioral strategies have been developed primarily for specialty settings and treatment programs where they can be effectively delivered, screening and brief intervention counseling has been developed for use in primary care settings, with a focus on treatment referral when necessary. Medication use in these nonspecialized settings and in a spectrum of patients including nondependent individuals is a recent phenomenon.

Research is needed to address the optimal use of medication therapy for the treatment of alcohol use disorders and for treating the broader spectrum of unhealthy alcohol use, from non-dependent risky drinking to alcohol dependence. This is especially true given the major scientific advances in pharmacotherapy that have been made over the past 60 years. To improve access to effective medication therapy, research also should explore the use of these medications in a range of health care settings. To optimize medication treatment outcomes, practitioners need to assess both the appropriate level of counseling (from minimal to more intensive) and the appropriate methods to enhance medication adherence for individual patients. The development of medications to address the spectrum of unhealthy alcohol use across the broad range of health care settings has the potential to maximize benefits for future patients.

After reviewing the medications currently approved for alcohol dependence and new medications being investigated, this article will outline ways to optimize treatment outcomes through patient–treatment matching and increased treatment adherence and review potential uses of medications for nondependent hazardous drinkers, including the use of medications in primary care settings.

## Medications for Alcohol Dependence

The Food and Drug Administration (FDA) has approved four medications for the treatment of alcohol dependence: disulfiram (Antabuse^®^), oral naltrexone, extended-release naltrexone (Vivitrol^®^), and acamprosate (Campral^®^). Topiramate, a medication used to treat epilepsy and migraine, has demonstrated evidence in two clinical trials of alcohol dependence, and a number of other promising medications are being studied. For detailed information about mechanisms, risks, and benefits of approved medications and those on the horizon, please see Krishnan-Sarin (2008). (For specific reviews: disulfiram [Malcom et al. 2008], oral naltrexone [Pettinati et al. 2006], injectable naltrexone [Swainston Harrison et al. 2006], acamprosate [Scott et al. 2005; Mason and Crean 2007], topiramate [Johnson and Ait-Daoud 2010] and the product information for each medication). The National Institute on Alcohol Abuse and Alcoholism’s (NIAAA’s) *Clinician’s Guide* provides practical information about prescribing medications for alcohol dependence (NIAAA 2007) and covers a range of considerations (e.g., concurrent counseling, length of treatment, mechanisms, contraindications, precautions, adverse events, drug interactions, and usual adult dosage).

Disulfiram, the first drug approved for the treatment of alcohol dependence, and still one of the most commonly used agents, produces an aversive interaction with alcohol by interfering with the metabolism of alcohol. During alcohol metabolism, alcohol is converted to acetaldehyde, which then is broken down by the enzyme aldehyde dehydrogenease. Disulfiram inhibits this later step, leading to a build up of acetalydehyde and results in aversive effects such as nausea, vomiting, palpitations, and headache. Ordinarily, the negative consequences of alcohol consumption (e.g., health problems) are delayed and are uncertain (e.g., your significant other may or may not become angry with you; the police may not apprehend you for drunk driving). The knowledge of the potential disulfiram alcohol interaction, however, can make the consequences of drinking certain and immediate and thereby support a person’s motivation to avoid drinking, and the actual reaction may limit the amount consumed if abstinence is violated. Medication compliance can be a problem, however, and disulfiram is most effective when provided with supervised administration by a significant other or health care provider (Krampe and Ehrenreich 2010).

Naltrexone is an opiate antagonist that primarily blocks μ-receptors with more variable occupancy of δ-receptors at the standard dose of 50 mg daily (Weerts et al. 2008). In laboratory studies, naltrexone has been shown to reduce the number of drinks consumed (Anton et al. 2004; Krishnan-Sarin et al. 2007; O’Malley et al. 2002). In clinical trials, naltrexone reduced the percentage of heavy drinking days (Pettinati et al. 2006). Recent meta-analyses have indicated that oral naltrexone has modest efficacy over 3 months on preventing relapse to heavy drinking, return to any drinking, and medication discontinuation (Srisurapanont et al. 2005). The standard dose is 50 mg daily, but a multisite study demonstrated that 100 mg daily also was effective when combined with medical management (Anton et al. 2006).

Extended-release naltrexone, a formulation that only requires a monthly injection, holds the potential to minimize problems with medication adherence. In a 6-month trial, 64 percent of participants received all 6 months of double-blind medication, translating into daily coverage for the entire treatment period (Garbutt et al. 2005). Naltrexone was significantly more effective in reducing the rate of heavy drinking than placebo, an effect most pronounced in those who had achieved abstinence prior to receiving the first injection. In the subset of those who were abstinent for at least 4 days prior to random assignment, extended-release naltrexone also significantly improved continuous abstinence rates (O’Malley et al. 2007). Specifically, 32 percent of those receiving extended-release naltrexone (380 mg) remained abstinent over 6 months compared with 11 percent of those receiving placebo.

The primary adverse effects of naltrexone, whether oral or injectable, are nausea followed by headache and dizziness. Patients with significant liver disease are not candidates for naltrexone nor are patients who require opiate medications for pain control. Acute pain control requires alternatives to opioids. To avoid precipitating an opioid-withdrawal syndrome, patients should be free of opioids for 7 to 10 days before beginning naltrexone. If extended-release naltrexone is administered subcutaneously rather than as an intramuscular gluteal injection, the likelihood of severe injection-site reactions may increase (http://www.vivitrol.com/pdf_docs/prescribing_info.pdf).

Acamprosate, available in oral delayed-release tablets (Campral^®^), was approved for use in the treatment of alcoholism in the United States in 2004, following extensive use in many other countries. Acamprosate is believed to normalize the balance between excitatory and inhibitory pathways altered by chronic alcohol consumption (Littleton and Zieglgansberger 2003), although the actual mechanism of action is uncertain. Using combined data from three European studies that were the basis of the approval of acamprosate in the United States, Kranzler and Gage (2008) found that acamprosate improved rates of continuous abstinence, percent days abstinent, and time to first drink. Two studies conducted in the United States did not find overall efficacy for acamprosate (Anton et al. 2006; Mason et al. 2006); however, the methods of these studies differed in substantial ways from the European studies. Notably, 90 percent of patients in the European acamprosate clinical trials received inpatient detoxification, compared with only 2.3 percent and 7.7 percent of those in U.S. trials (Mason and Crean 2007).

One of the strengths of acamprosate is its side-effect profile; the most common side effects are gastrointestinal in nature. Acamprosate can be used in patients with moderate liver disease but is contraindicated in patients with severe renal impairment, and dose reductions are recommended for those with mild-to-moderate levels of renal impairment.

Topiramate, an anticonvulsant, is hypothesized to have beneficial effects on drinking by facilitating functioning of the neurotransmitter γ-aminobutyric acid (GABA) and antagonizing glutamate activity. Two placebo-controlled trials (Johnson et al. 2003, 2008), including a multisite study, have demonstrated the efficacy of topiramate in very-heavy-drinking alcohol-dependent patients who were not required to be abstinent prior to starting treatment. In these trials, therapists used brief behavioral compliance enhancement therapy to enhance medication adherence and provide support for patients who worked on their personal goals for their drinking. Patients also reduced cigarette smoking, which suggests a potential side benefit of using topiramate to treat alcohol-dependent smokers (Johnson et al. 2005).

Topiramate requires very gradual dose escalation. The most common adverse events include cognitive dysfunction, abnormal sensations (e.g., numbness, tingling), and anorexia and taste abnormalities. Additional rarer serious adverse events have been identified, such as metabolic acidosis, acute myopia, and secondary narrow-angle glaucoma. The optimal dose for alcohol dependence has yet to be established and may be lower than that the target dose of 300 mg per day tested in prior research.

### New Medications on the Horizon

Currently available pharmacotherapies only have modest effects, which has spurred efforts to identify treatment responders, new medications, treatment combinations, and methods to enhance adherence. As reviewed by Krishnan-Sarin and colleagues (2008), several other medications show some clinical evidence of efficacy.

Numerous studies have tested selective serotonin reuptake inhibitors (approved for depression), often with disappointing results including counter-therapeutic effects among patients with early-onset alcoholism. However, studies show that these medications (e.g., sertraline) may be efficacious among individuals with later-onset alcoholism (Kranzler et al. 1996; Pettinati et al. 2000) or in combination with naltrexone for patients with major depression (Pettinati et al. 2010). In contrast, ondansetron (a selective serotonin-3 [5HT_3_] antagonist approved for nausea) shows some efficacy for reducing heavy drinking among patients with early-onset or Type-B alcoholism (Kranzler et al. 2003; Johnson et al. 2000).

Medications targeting GABA and glutamate systems show promise as treatments for acute and protracted alcohol withdrawal and for relapse prevention. Treatment with baclofen (a GABA_b_ receptor agonist [i.e., it binds with the GABA_b_ receptor] used for muscle spasticity) has been found to reduce symptoms of alcohol withdrawal, and a placebo-controlled study of 84 alcohol-dependent patients with cirrhosis yielded promising results (Addolorato et al. 2007). However, a recent placebo-controlled study in 121 patients did not find an advantage of baclofen over placebo on measures of drinking, although baclofen was associated with reduced anxiety (Garbutt et al. 2010). Additional efficacy studies will need to address whether individuals with more severe dependence or greater anxiety may benefit from this medication. Gabapentin, an anticonvulsant, also shows promise for alcohol withdrawal and for improving drinking outcomes in early treatment among individuals with high alcohol-withdrawal symptoms (Anton et al. 2009) or individuals with comorbid insomnia (Brower et al. 2008).

Given the role of dopamine in the maintenance of alcohol dependence, drugs that have direct effects on dopamine through either partial agonism (e.g., aripiprazole) or through antagonist effects (e.g., olanzapine, quetiapine) have been investigated as candidates for alcoholism treatment. A multisite study did not find an overall advantage of the atypical antipsychotic aripiprazole over placebo on the primary outcomes, although some secondary outcomes suggested that studies at lower doses would be worthwhile (Anton et al. 2008*a*). A smaller, single-site, placebo-controlled study did not show a benefit of olanzapine, and, although not statistically significant, discontinuation of treatment was higher in the group receiving active medication compared to the group receiving placebo. The anti-psychotics all have important adverse events that may limit the potential of these agents for treating alcohol dependence.

There has been considerable enthusiasm about the potential of rimonabant, a cannabinoid receptor 1 antagonist, based on preclinical research showing that it reduced alcohol drinking. However, psychiatric adverse events noted in obese patients, a negative human alcohol self-administration study (George et al. 2009), and a negative clinical trial in individuals with alcohol dependence (Soyka et al. 2008) have ruled out this particular agent for the treatment of alcohol dependence.

Many alcohol-dependent individuals also smoke cigarettes, and researchers have investigated the potential role of the nicotinic acetylcholine receptor (nAChR) system as a factor in both addictive behaviors (for a review, see Chatterjee and Bartlett 2010). Nicotinic compounds, including agonists, partial agonists, and antagonists, currently are under investigation for the treatment of alcoholism. Human laboratory studies have shown that mecamylamine, a nonselective nAChR antagonist approved for hypertension, can reduce alcohol preference and the stimulating effects of alcohol in healthy study participants (Blomqvist et al. 2002; Chi et al. 2003; Young et al. 2005). Laboratory studies also have shown that varenicline, a partial agonist approved for smoking cessation, can reduce craving and drinking in smokers who drink heavily (McKee et al. 2009). A preliminary study among smokers receiving varenicline for smoking cessation found that it significantly reduced heavy drinking (compared with a placebo) during an extended pretreatment period (Fucito et al. in press). Studies are ongoing to evaluate the efficacy of these two compounds in clinical trials of alcohol-dependent patients.

Researchers also are studying agents that may address the relationship between stress and alcohol consumption. Prazosin, an α-1 adrenergic antagonist that is effective in treating posttraumatic stress disorder (PTSD), has shown preliminary efficacy in a small pilot study with 24 alcohol-dependent patients without PTSD (Simpson et al. 2009). Other targets for new treatments are receptors for stress-related neuropeptides, including corticotrophin releasing factor (CRF), neuropeptide Y (NPY), substance P, nociceptin (George et al. 2008; Heilig and Egli 2006), and inhibitors of ALDH-2 (Overstreet et al. 2009).

## Optimizing Outcomes by Patient–Treatment Matching

Research is being done in an attempt to identify predictors of patient response to FDA–approved treatments. In a secondary analysis of a U.S. acamprosate trial, patients with a strong commitment to abstinence benefited from acamprosate (Mason et al. 2006). However, several hypothesized predictors of acamprosate response, including high physiological dependence, late age-of-onset, and serious anxiety symptoms, did not predict differential response in a pooled analysis of data from seven placebo-controlled trials. A secondary analysis of baseline trajectories of drinking in the Combining Medications and Behavioral Interventions for Alcoholism Study (COMBINE), the largest study of pharmacotherapy to date, found that individuals who achieved 14 or more days of abstinence may not be good candidates for acamprosate whereas those who were frequent drinkers but did not attain extended abstinence may benefit (Ralitza et al. in press). With regard to naltrexone, several studies, but not all, have suggested that family history of alcoholism (Krishnan-Sarin et al. 2007; Monterosso et al. 2001; Rohsenow et al. 2007) and a variant of the opioid receptor, μ-1 (OPRM1) may predict differential benefit (Anton et al. 2008*b*; Oslin et al. 2003). In the COMBINE study, people with “Type A” alcohol dependence (i.e., fewer co-morbid psychiatric and substance abuse disorders) responded well to naltrexone (Bogenschutz et al. 2009). Because primary care providers may feel more comfortable managing less complicated patients, this is an encouraging finding. In the end, the promise of personalized medicine will depend on the identification of reliable predictors of differential treatment response.

## Optimizing Outcomes by Increasing Adherence

Poor adherence to prescribed medications c limit an a treatment’s effectiveness. As a result, research has investigated predictors of adherence and methods for enhancing adherence. One of the best predictors of future behavior is past behavior. In the case of medication compliance, self-reported problems with adherence characterized as purposeful nonadherence (e.g., stopping medication early due to either feeling better or worse) predict medication compliance and treatment outcome (Toll et al. 2007). Randomized controlled trials of interventions to enhance adherence to medications for disorders other than addictions suggest that several short-term interventions are associated with improved adherence and outcome (Haynes 2008). These included providing patients with written instructions, simple counseling about adherence (e.g., that all the medications should be taken), and personal phone calls. For long-term treatments, the meta-analysis conducted by Haynes (2008) indicated that simplifying the dosage regimen and more complex interventions, including combinations of interventions, were nominally effective, with the common feature being ongoing contact emphasizing adherence.

Researchers also have developed brief interventions to support adherence to alcoholism medications. Common features of these interventions include emphasizing the importance of adherence, providing positive feedback for good adherence, and problem-solving difficulties with adherence. The Medication Management Intervention (Pettinati et al. 2004, 2005), BRENDA^1^ (Volpicelli et al. 2001), and Brief Behavioral Compliance Enhancement Treatment (BBCET) (Johnson et al. 2003) all incorporate these components. Behavioral interventions also can be more intensive, including providing contingent financial incentives for adherence and family interventions. Pharmacological solutions include reducing adverse events (Rohsenow et al. 2000) and the development of formulations that require less frequent administration, such as extended-release naltrexone.

## Medication Use for Nondependent Hazardous Drinkers

Currently, research has evaluated alcoholism medications primarily in alcohol-dependent populations. Many individuals, however, drink at harmful levels but do not meet the criteria for dependence and may benefit from medications to augment counseling approaches used with this subgroup of drinkers.

### Young Adults

Because young adults are less interested in quitting drinking than in reducing their drinking, interventions to help them moderate their alcohol consumption may be particularly useful (Epler et al. 2009). Brief motivational interventions, such as the Brief Alcohol Screening and Intervention for College Students (BASICS), incorporate personalized normative feedback, assess interest in moderating drinking, and provide behavioral strategies for avoiding alcohol-related harm. Witkiewitz and Marlatt (2006) reported modest effects for these interventions when compared with no treatment and with alcohol education. The possibility of combining medications with brief motivational interventions for young adults warrants further investigation. Naltrexone, for example, may be well suited to this population because it can reduce drinking even in the absence of alcohol counseling (O’Malley et al. 2009; Tidey et al. 2008) and targeted administration of naltrexone in anticipation of high-risk situations significantly reduced drinking among problem drinkers (Kranzler et al. 2009). A preliminary open-label study of naltrexone and BASICS in young adults suggests that this approach is associated with reductions in heavy drinking and alcohol-related consequences (Leeman et al. 2008).

### Reducing Drinking

Regardless of age, many individuals with alcohol dependence, particularly those with less severe problems, would prefer to reduce their drinking rather than seek total abstinence, and low severity of alcohol dependence is one of the characteristics that predicts recovery from alcohol problems, as evidenced by moderate drinking (Humphreys et al. 1995). As a result, medications that reliably reduce the risk of heavy drinking would likely enhance treatment seeking, especially among individuals with less severe problems. In this regard, topiramate and naltrexone show potential for a subset of patients. However, the field needs to identify which patients achieve and maintain nonhazardous drinking with these medications and better medications that have this effect for a broad spectrum of patients. Finally, studies of medications to reduce hazardous drinking should incorporate behavioral interventions intended to realize reduced hazardous drinking as the treatment goal (for a review of behavioral interventions to promote moderate drinking, see Witkiewitz and Marlatt 2006). In addition, NIAAA has released an interactive Web-based program, *Rethinking Drinking,* that provides individuals with empirically supported information and tools for reducing drinking (http://rethinkingdrinking.niaaa.nih.gov/).

## Medication Use in the Treatment of Unhealthy Alcohol Use in Primary Care Settings

The rapid progress in the development of medications to treat alcohol dependence, although impressive, has resulted in a relatively slow adaptation of these new treatments. In 2007, the percentage of Veterans Administration patients with alcohol use disorders who received pharmacotherapy was 3 percent (Harris et al. 2010). Among patients seen in the past year in 128 Veterans Health Administration facilities, the rates ranged from 0 to 20.5 percent among those who received specialty care and from 0 to 4.3 percent among those who did not receive specialty care. A number of obstacles have hindered medication use in alcohol dependence treatment programs, including lack of knowledge and availability of medical staff who can prescribe. However, researchers have identified the following factors associated with the adoption of medication use: organizational characteristics, such as accreditation; the presence of staff physicians; and the availability of detoxification (see the sidebar by LaPaglia on p. 305).

Primary care providers are well suited to address a wide variety of behavioral problems in their patients and routinely manage chronic diseases with a combination of counseling and medication management. Using primary care–based prevention strategies to address behaviors such as overeating and smoking, practitioners already routinely screen for conditions such as high cholesterol, hypertension, and cancer and treat a full range of chronic conditions such as diabetes and asthma. Similar clinical management strategies for unhealthy alcohol use and alcohol use disorders have been developed. However, despite convincing data supporting the value of evidence-based screening techniques, brief interventions, and medication approaches, primary care settings rarely use these tools (D’Amico et al. 2005). In an effort to address this situation, the Institute of Medicine (2005) strongly endorsed the notion that primary care providers should have a greatly enhanced role in identifying and managing substance use problems in their patients as part of a strategy to improve access to care for individuals with substance use disorders.

With regard to alcohol, a single question about how often the patient exceeds the daily maximum drinking limits (i.e., more than four drinks for men and more than three drinks for women) in the prior year can be effectively used to screen for unhealthy alcohol use (Willenbring et al. 2009). The NIAAA clinician’s guide, *Helping Patients Who Drink Too Much*, provides practical advice about how to follow up with individuals who screen positively for excessive drinking (NIAAA 2009). The guide recommends that clinicians evaluate the potential use of alcoholism medications as a treatment component for patients who screen positively for excessive drinking.

### Studies of Medication Use in Primary Care

Evidence supporting the potential use of alcoholism medications in primary care settings derives from studies conducted in such settings and studies that compared specialty care with primary care models of counseling. These studies provide clues to the nature and amount of behavioral counseling needed to accompany pharmacotherapy. Some studies address both of these questions (or do not separate the questions), whereas others address one or the other. Most studies have not enrolled primary care patients but have evaluated primary care models of treatment provided by medical providers who are not alcoholism specialists in research settings.

In an initial study examining the effectiveness of naltrexone in combination with a primary care model of care, 197 alcohol-dependent participants were treated with naltrexone for 10 weeks in combination with cognitive–behavioral therapy (CBT) provided by an alcoholism specialist or in combination with primary care management (PCM) provided by a primary care practitioner (O’Malley et al. 2003). Treatment response was similar at the end of 10 weeks, with 84.1 percent (74 of 88) of the PCM patients and 86.5 percent (77 of 89) of the CBT patients avoiding persistent heavy drinking. Among those who responded to a primary care model, continued treatment with naltrexone for 6 months significantly helped sustain gains. Among those receiving CBT, maintenance of response remained relatively high and continued naltrexone did not improve this outcome significantly over placebo.

The COMBINE Study (Anton et al. 2006) tested the efficacy of medications for alcoholism in the context of a medical management model of counseling in contrast to an approach in which patients received medical management and specialist counseling. In this study, eight groups of recently alcohol-abstinent individuals with diagnoses of primary alcohol dependence based on the *Diagnostic and Statistical Manual of Mental Disorders, Fourth Edition* received medical management with 16 weeks of naltrexone (100 mg per day) or acamprosate (3 g per day), both naltrexone and acamprosate, and/or both placebos, with or without a combined behavioral intervention (CBI). A ninth group received CBI only (no pills).

The medical management intervention consisted of up to nine sessions over 16 weeks with a health care professional (e.g., a nurse, physician’s associate, nurse practitioner, or physician). Following an initial 45-minute interview, subsequent appointments were approximately 15 minutes long. The approach included monitoring of drinking, support and encouragement, establishing a plan for medication adherence, monitoring and problem solving adherence issues, and advice to attend support groups (e.g., Alcoholics Anonymous).

The results indicated that patients who received naltrexone plus medical management, CBI plus medical management and placebos, or naltrexone and CBI plus medical management had higher percentages of days abstinent (80.6, 79.2, and 77.1 percent, respectively) than those receiving placebos and medical management only (75.1 percent). Naltrexone also reduced the risk of having a heavy-drinking day, but this effect was most evident in those receiving medical management but not CBI. Acamprosate showed no significant effect on drinking versus placebo, either by itself or with any combination of naltrexone, CBI, or both. These results suggest that health care providers could use a primary care model of counseling with pharmacotherapy to improve treatment outcomes.

Consistent with the findings in COMBINE, O’Malley and colleagues (2007) demonstrated that a 50-mg daily naltrexone regimen in combination with medical management also was effective in a rural Alaskan environment among alcohol-dependent individuals (primarily Alaska natives). Naltrexone significantly improved abstinence rates and decreased rates of alcohol-related consequences over the course of the 16-week treatment. Given that access to specialty care often is limited in rural communities, the potential of incorporating pharmacotherapy into primary care practice could help reduce important health disparities resulting from limited access to treatment.

Oslin and colleagues (2008) completed the only study that has evaluated the intensity of interventions that primary care providers might use. In this 24-week study, participants received naltrexone or placebo and one of three psychosocial interventions. All participants attended nine brief medication visits with a physician for safety monitoring, brief review of drinking, and dispensing of medications. One group only received these doctor visits. A second group received up to 18 additional counseling sessions with a nurse practitioner based on the BRENDA model (Volpicelli et al. 1997), which includes aspects of motivational counseling and specifically focuses on adherence to treatment, progress made toward reducing alcohol consumption, problem-solving, and self-change strategies. The third group received up to 18 individual CBT sessions with a clinical psychologist or social worker.

The results favored CBT compared with the two other less intensive treatments. The differences between the BRENDA and the doctors-only groups were not significantly different. The effect of naltrexone was not significant and did not vary by the type of psychosocial intervention, although the sample size was too small to detect anything other than very large interaction effects. Medication adherence was relatively low (50 percent took medication on 80 percent of days over the 24 weeks of therapy) and may have been related to the relatively longer duration of the study and the use of the 100-mg dose. Medication adherence was associated with better outcomes, irrespective of medication condition.

Extended-release naltrexone appears to be well suited for use in primary care settings. Skilled medical personnel are required to administer extended-release naltrexone with an intramuscular gluteal injection; many specialty programs do not have access to needed medical care providers. Moreover, the efficacy studies of extended-release naltrexone used BRENDA counseling, albeit the frequency of appointments may have exceeded that likely to occur in primary care. Future studies should evaluate the efficacy of once-a-month extended-release naltrexone with less frequent counseling and in patients recruited through primary care sites.

As reviewed by Mason and Crean (2007), the European studies of acamprosate typically enrolled participants who had completed inpatient detoxification and then received standard care as outpatients. The treatment outcomes, including time to first day of drinking or cumulative abstinence duration, were very similar whether patients received brief interventions or intensive treatments including relapse prevention therapy, individual therapy, group therapy, or family therapy in addition to acamprosate (Pelc et al. 2002; Soyka et al. 2002).

One of the few medication trials actually conducted in primary care sites (Kiritze-Topor et al. 2004) compared standard care to standard care with acamprosate among 422 alcohol-dependent patients recruited and treated for 1 year in general practices. Patients treated with acamprosate and standard care showed significantly greater improvement, with 64 percent reporting no alcohol-related problems for 1 year compared with 50.2 percent of those receiving standard care alone. Although the study physicians had prior experience treating alcoholism and had participated in at least one clinical trial, the general conclusion from this study was that general practitioners could effectively use acamprosate to manage alcohol dependence. The low loss to follow-up of 17 percent over 1 year highlights a potential advantage of treating patients in primary care, where patients have ongoing relationships with their providers compared with specialty care programs, where drop-out rates are substantially higher.

Recent studies of continuing-care interventions suggest that interventions of a year or longer and treatments that are less burdensome can promote sustained engagement and positive effects (McKay 2006). As discussed above, the use of medications by primary care providers may be a viable approach to providing low-intensity longer-term treatment. Patients also may be open to this approach. In a survey of medially hospitalized patients with alcohol dependence (Stewart and Connors 2007), 66 percent agreed that they would like to receive a medication that would help prevent drinking, and 32 percent were interested in primary care treatment.

In summary, the research literature supports the effectiveness of medications, such as naltrexone, in combination with models of care that primary care providers or medical professionals associated with specialty alcohol programs could use. Several published manuals (NIAAA 2009; Pettinatti et al. 2004, 2005; Volpicelli et al. 2001) are available that detail the specifics of these approaches. In prior research, these primary care interventions involved brief but frequent appointments. As discussed earlier, frequent contact is likely to enhance medication adherence and contribute to the effectiveness of medications. However, researchers have not yet established the tradeoff between decreasing the frequency of follow-up in conjunction with primary care counseling and the effectiveness of medication treatments for alcoholism. Even research on injectable naltrexone, a once-a-month preparation, was evaluated in conjunction with 12 sessions over 6 months. As a result, additional research is required in order to guide clinical practice about the minimal frequency of counseling, but this should not prohibit the use of FDA-approved medications in these settings.

### Implementing Medication Use in Primary Care Settings

In the management of both acute and chronic conditions, physicians and other medical professionals often need to consider carefully when to suggest medication treatment to individual patients. Typically, the decision to recommend medication treatment relies on a combination of an assessment of the evidence to support a particular therapy for a specific condition and clinical judgment concerning whether an individual patient is appropriate for that treatment based on a variety of patient-and disease-specific features. Clearly, such decisions are best arrived at using a patient-centered approach involving patient education, preferences, and mutual decisionmaking. Even when medication therapy has a clear evidence base in a given clinical situation, patients and their providers may identify a variety of reasons why a specific therapy may or may not be used. Beyond this, research often demonstrates that there are certain patient subgroups for whom a specific therapy may or may not be particularly effective. These subgroups may be identifiable based on clinical, demographic, genetic, or social features that all may play a major role in the decision process regarding medication use. With the availability of several FDA-approved medications, a provider may recommend a trial with a new medication should an individual patient not respond to the first medication tried.

The implementation and widespread use of medications to treat alcohol problems faces a unique set of barriers in primary care. Although primary care providers are proficient at prescribing a wide variety of medications, they generally are unfamiliar with medications for treating alcohol problems other than those used to treat alcohol withdrawal. Indeed, a growing body of research to support basic screening methods, brief interventions, and especially medication therapy has yet to have a major impact on how primary care providers care for individuals at risk for or with alcohol problems (D’Amico et al. 2005). The results of studies on how to enhance the use of screening and brief intervention, however, may inform how to promote medication treatments for alcohol problems in primary care. For example, in one study, practice-based provider education and quality improvement activities resulted in a 65 percent screening rate (compared with 24 percent in control practices) and a 51 percent counseling rate (versus 30 percent in control practices) (Rose et al. 2008). In addition, the success of strategies to implement screening and brief-intervention practices in primary care appears to rely on a variety of complex provider and organizational characteristics (Babor et al. 2005). Understanding and addressing these characteristics may be particularly important if these medications are to gain acceptance in primary care. Finally, “marketing“ strategies shown to be helpful with the implementation of brief intervention counseling, such as telemarketing and academic detailing (Funk et al. 2005), may be particularly useful in enhancing primary care physicians’ use of medications for treating alcohol problems. Future research should carefully examine the effectiveness of these and other approaches to improving the extent to which primary care physicians can be prompted to use effective medications when appropriate to treat their patients with alcohol problems.

## Summary

Identifying and treating people with alcohol use disorders remains a challenge. With the advent of pharmacotherapy and models of counseling appropriate for use in primary care settings as well as in specialty care, clinicians have new tools to manage the spectrum of alcohol problems across the spectrum of patients and settings. By extending the continuum of care to primary care settings, many people who do not currently receive specialty care may have increased access to treatment. In addition, primary care providers, by virtue of their ongoing relationships with patients may be able to provide continuing care interventions. Medication use with hazardous drinkers who may not be alcohol dependent may promote reduced drinking and likely will reduce the burden of excessive drinking.

## Footnotes

1 The framework for BRENDA is based in the biopsychosocial model of addiction and consists of six stages: the clinician’s (1) biopsychosocial evaluation of the patient, (2) report of that assessment back to the patient, (3) empathy for the patient’s situation, (4) needs identification by both patient and clinician, (5) direct advice to the patient on how to meet those needs, and (6) assessment of the patient’s reaction to that advice as well as any necessary adjustments to the treatment plan.
