# Supplementary material for: Medications for Unhealthy Alcohol Use: Across the Spectrum
Source: Alcohol Res Health. 2011;33(4):300–12. (PMC3860540)
Supplement: Related texts

# Sub article: Challenges and Solutions of Adding Medications Treatment to Specialty Addiction Treatment Programs: A Review With Suggestions

**Published:** 2011

**Authors:** Donna LaPaglia

Slow diffusion of evidence-based innovations is a common occurrence in health care. Rogers (2003) documented the lag that exists between proven scientific benefits and their adoption into formal practice. This gap is very pronounced in addictions treatment, despite documented evidence of therapies that show promise in treating substance use disorders (Lamb et al. 1998; McGovern et al. 2004; Sorenson and Midkiff 2002). This widely acknowledged gap occurs for psychotherapeutic interventions as well as established pharmacotherapies.

A multitude of factors are thought to influence the substance abuse treatment community’s ability and/or willingness to incorporate these practices into routine care. This sidebar focuses specifically on the adoption of medication-based approaches for the treatment of alcohol dependence, describes the historical context and the environmental milieu of current addictions treatment, and makes recommendations for the successful implementation of medications use in addiction treatment programs.

Data from the National Survey on Drug Use and Health indicate that in 2007, 7.8 percent of people over the age of 12 years (approximately 19.3 million people) required treatment for an alcohol use problem (Substance Abuse and Mental Health Services Administration 2009). Of 19.3 million people with an alcohol use problem, only 8.1 percent (1.6 million) actually received treatment at a specialty care setting. Mark and colleagues (2009) found that 720,000 prescriptions were filled in 2007 for alcoholism medication.

Medication-assisted treatment accounts for a small percentage of ongoing substance abuse treatment in this country. With a vast majority of the substance-using population not reaping the benefits of addiction medications, it is necessary to examine the historical beginnings of addictions treatment to inform adoption recommendations.

Because of social stigma, addictions treatment grew in isolation from mainstream medical care (Guydish 2003; White 1998), with recovering peers ministering to each other out of necessity. The system of care that evolved carried with it a “personal” focus with peer teachings spread by word of mouth. These teachings and the surrounding attitudes and belief systems emphasize self-reliance and the belief that healing can take place solely within the community of addicted people and that no medical intervention is necessary. Addiction treatment programs sprang forth from Alcoholics Anonymous (Alcoholics Anonymous 1976) and other step-based movements. The resulting system of care possesses, at its core, a philosophical belief that total abstinence is gained not through the use of medication to treat alcohol dependence but instead through blood, sweat, and personal tears working through the 12 steps.

The most recent Federal data indicate that non-medical personnel, many of whom possess personal 12-step recovery histories, deliver the majority of alcoholism treatment in this country in specialty care settings. These treatment programs differ widely in organizational structure, source of payment, services offered, leadership characteristics, staff credentials, presence of medical personnel, program size, and patient characteristics.

Data from these specialty care settings indicate that adoption of medication for the treatment of alcohol disorders is uncommon in both the public and private sector (Ducharme et al. 2006). An examination of public reimbursement as reported by the National Conference of State Legislatures (2008) indicates that Medicaid coverage of substance abuse medications is not common among States and that it is an option not a requirement (Gelber 2008).

Accordingly, factors that may positively influence the adoption of medication use should target State regulatory structures, availability of medical staff, community linkages, and curricula of alcohol and drug training programs as well as graduate psychology programs. Focus on the following areas may increase a program’s readiness for the adoption of medication use:

- Increase State agencies’ understanding of the benefits related to medication-assisted therapies for addiction in an effort to increase acceptance for public funding.
- State licensing requirements for programs could be amended to require greater availability of medical staff and credentialed counselors, given that the presence of medical personnel is key to the adoption of medication use, as is the presence of counselors with higher educational attainment (i.e., Masters level or higher) (Knudsen et al. 2005).
- Formal linkages between specialty treatment providers and primary care physicians could allow for the flow of information, expertise (clinical and medical), and support, thus enhancing the experiential base of both providers.
- Graduate psychology programs, addiction psychiatry fellowships, and drug and alcohol programs should include evidence-based treatments in curriculum and internship training opportunities. Currently, degree programs often neglect evidence-based treatments, may not offer addictions course work, and may not offer opportunities for students to develop competence in any empirically validated treatments, psychosocial or pharmacotherapeutic (Crits-Christoph et al. 1995; Miller and Brown 1997).
- Most substance abuse counselors indicate that they do not use scientific journals to inform practice (Miller 1987*b*; Sobell 1996). As a result, dissemination of information about medications to program members, from directors to line staff, should occur through publications in trade journals and newsletters, continuing education course work, professional meetings, and face-to-face interaction (workshops). For the education to be effective it must be written or translated into everyday language using actual case examples of programs successfully adopting medications. This approach is personal and positive and can be quite powerful for substance abuse counselors with recovery histories.
- With the accumulating evidence base for motivational enhancement strategies (Carroll et al. 2006) and more widespread experience with these interventions, medication adoption no longer challenges program treatment philosophy. Instead, it may be viewed as supporting clients who are motivated to achieve and maintain continued abstinence.
- Ongoing consultation, supervision, and feedback are useful for programs adopting and maintaining the practice of treating alcohol disorders with medication.

Agencies that have decided to utilize medications for the treatment of alcohol disorders should consider the following suggestions for successful implementation and maintenance:

- Programs can offer incentives for attendance at training sessions (e.g., time off with pay), food and prizes at the training event, bonuses, or promotions contingent on achieving a level of competence with the use of the pharmacotherapy (Carise et al. 2002).
- Programs should develop plans for incorporating medications into their existing practice. Process improvement methods, such as the NIATx Way (Langley et al. 1996), provide a potential tool for doing this. Process improvements allow agencies to make major changes by tackling one small project in a short amount of time (2- to 4-week turnaround). The steps for setting up a change project (with examples for addressing use of pharmacotherapy) are as follows: (1) Gather data for the indicator you wish to change (e.g., number of alcohol-dependent clients within your program currently treated with medication); (2) determine the target population (e.g., alcohol-dependent clients with no prior medication attempts and prior psychosocial treatment failures); (3) establish a clear aim (e.g., greater engagement in treatment in the targeted population); (4) select a change leader (i.e., a positive, energized person who has the ability to leverage and or interact with all levels of the organization); and (5) create a team (including employees from all levels of the organization) responsible for developing and implementing the change. As one example, the team might decide to implement a tickler reminding the physician to discuss medications as part of the treatment plan review. At the end of the change period, the team analyzes the data and makes decisions (plan, do, study, act) based on the findings. This could include continuing the use of medication for clients with prior treatment failure or expanding the client base by including alcohol-dependent clients new to substance abuse treatment. The positive client results motivate the team to continue in this direction. Using this model creates natural “buy in;” employees are less likely to feel that incorporating medication adoption is a management-only decision because they had a hand in designing the program change.

## References

[b84-arh-33-4-300] Alcoholics Anonymous (1976). Alcoholics Anonymous: The Story of How Many Thousands of Men and Women Have Recovered From Alcoholism.

[b85-arh-33-4-300] Carise D, Cornely W, Gurel O (2002). A successful researcher-practitioner collaboration in substance abuse treatment. Journal of Substance Abuse Treatment.

[b86-arh-33-4-300] Carroll KM, Ball SA, Nich C (2006). Motivational interviewing to improve treatment engagement and outcome in individuals seeking treatment for substance abuse: A multisite effectiveness study. Drug and Alcohol Dependence.

[b87-arh-33-4-300] Crits-Christoph P, Frank E, Chambless DL (1995). Training in empirically validated treatments: What are clinical psychology students learning?. Professional Psychology, Research and Practice.

[b88-arh-33-4-300] Ducharme LJ, Knudsen HK, Roman PM (2006). Trends in the adoption of medications for alcohol dependence. Journal of Clinical Psychopharmacology.

[b89-arh-33-4-300] Guydish J, Sorensen JLA, Rawson RA, Guydishe J, Zwefen JE (2003). Introduction: Dissemination from practice to research. Drug Abuse Treatment Through Collaboration: Practice and Research Partnerships That Work.

[b90-arh-33-4-300] Knudsen HK, Ducharme LJ, Roman PM, Link T (2005). Buprenorphine diffusion: The attitudes of substance abuse treatment counselors. Journal of Substance Abuse Treatment.

[b91-arh-33-4-300] Lamb S, Greenlick MR, McCarty D (1998). Bridging the Gap Between Practice and Research: Forging Partnerships With Community-Based Drug and Alcohol Treatment.

[b92-arh-33-4-300] Langley GJ, Nolan KM, Nolan TW (1996). The Improvement Guide.

[b93-arh-33-4-300] Mark TL, Kassed CA, Vandivort-Warren R (2009). Alcohol and opioid dependence medications: Prescription trends, overall and by physician specialty. Drug and Alcohol Dependence.

[b94-arh-33-4-300] McGovern MP, Fox TS, Xie H, Drake RE (2004). A survey of clinical practices and readiness to adopt evidence-based practices: Dissemination research in an addiction treatment system. Journal of Substance Abuse Treatment.

[b95-arh-33-4-300] Miller WR, Brown SA (1997). Why psychologists should treat alcohol and drug problems. American Psychologist.

[b96-arh-33-4-300] Miller WR (1987b). Behavioral treatment research advances: Barriers to utilization. Advances in Behaviour Research and Therapy.

[b97-arh-33-4-300] National Conference of State Legislatures (2008). State Financing for Medication-Assisted Treatment: Results and Observations From a 50-State NCSL/AVISA Survey.

[b98-arh-33-4-300] Rogers EM (2003). Diffusion of Innovations.

[b99-arh-33-4-300] Sobell LC (1996). Bridging the gap between scientist and practitioners: The challenge before us. Behavioral Therapy.

[b100-arh-33-4-300] Sorensen JL, Midkiff EE (2000). Bridging the gap between research and drug abuse treatment. Journal of Psychoactive Drugs.

[b101-arh-33-4-300] Substance Abuse and Mental Health Services Administration (SAMHSA), Office of Applied Studies (2009). The NSDUH Report: Alcohol Treatment: Need, Utilization, and Barriers.

[b102-arh-33-4-300] White WL (1998). Slaying the Dragon: The History of Addiction Treatment and Recovery in America.
